# Case of resected small-cell neuroendocrine carcinoma of the extrahepatic bile duct

**DOI:** 10.1093/jscr/rjac020

**Published:** 2022-02-09

**Authors:** Hiroaki Sugita, Kazuya Maeda, Satoshi Nishikawa, Kenji Doden, Yasuo Hashizume

**Affiliations:** Department of Surgery, Fukui Prefectural Hospital, Fukui, Japan

## Abstract

Neuroendocrine carcinomas (NECs) arising from the extrahepatic bile duct (EHBD) are extremely rare, and their preoperative diagnosis is difficult. A small number of resected cases of EHBD NECs has been reported, and their prognosis is usually poor. A 62-year-old man presented with obstructive jaundice and liver disease. Radiological imaging revealed wall thickness and stricture of the distal common bile duct (CBD); however, lymph node or distant metastasis was not detected. Adenocarcinoma was detected on biopsy, and surgery was performed with a preoperative diagnosis of cholangiocarcinoma of the distal CBD. Pathological examination revealed adenocarcinoma of the CBD mucosa (20%) and NEC of the CBD wall (80%). The final pathological diagnosis was small-cell NEC of the EHBD. His post-operative course was good, and there was no recurrence for 4 months after surgery. Herein, we report a case of resected EHBD NEC and a literature review.

## INTRODUCTION

Neuroendocrine carcinomas (NECs) are tumors derived from neuroendocrine cells and are defined as poorly differentiated tumors with a high mitotic count (>20 per 10 high-power fields) and with Ki-67 index >20%. NECs can arise from different organs, but the extrahapatic bile duct (EHBD) is an extremely rare primary site of NECs [1]. A few cases of NECs in the EHBD have been reported, but most of them have a poor prognosis. Herein, we report a resected case of NEC of the EHBD.

## CASE REPORT

A 62-year-old man was referred to our hospital for evaluation of obstructive jaundice and liver disease. The patient presented with mild jaundice and pruritus of the skin. He had no specific medical, surgical or familial history of cancer. Upon admission, laboratory examinations revealed elevated levels of total bilirubin, transaminase and biliary enzymes. Serum levels of carcinoembryonic antigen and carbohydrate antigen 19-9 were within normal ranges.

**Figure 1:**
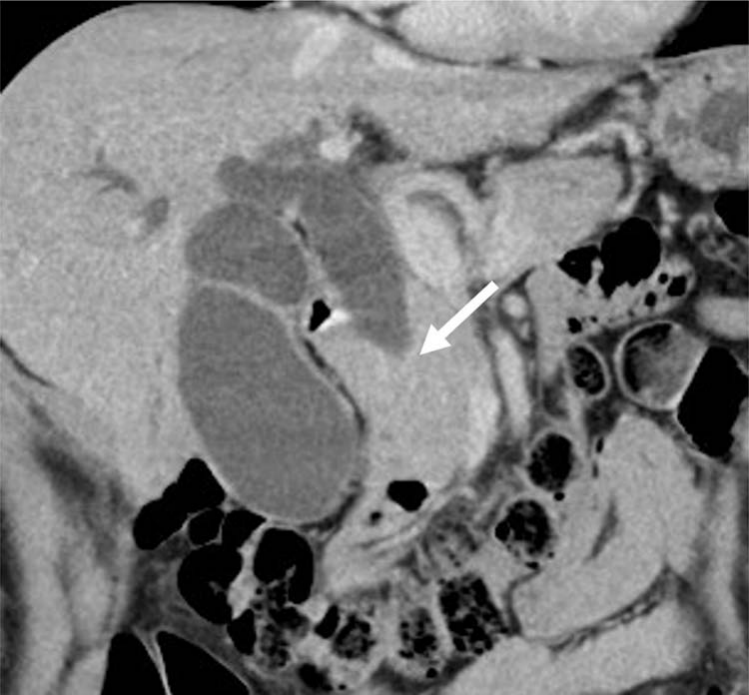
CT scan showed wall thickness and stenosis of the distal CBD (arrow).

**Figure 2:**
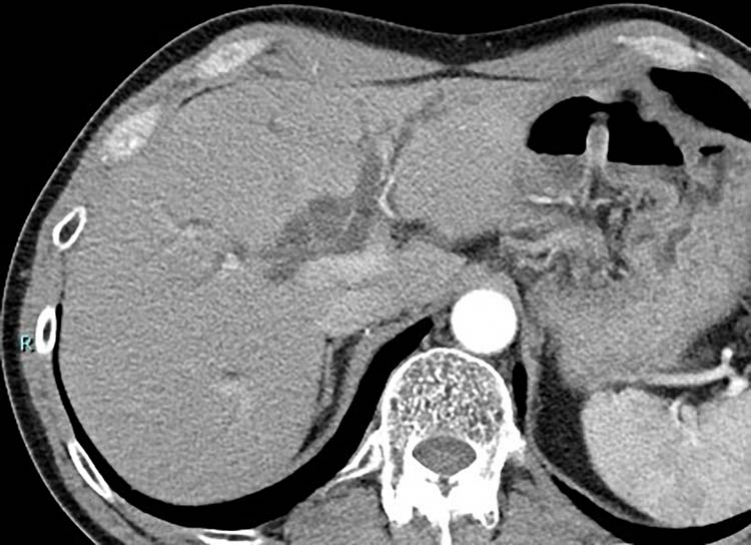
CT scan showed severe dilation of intrahepatic bile duct.

**Figure 3:**
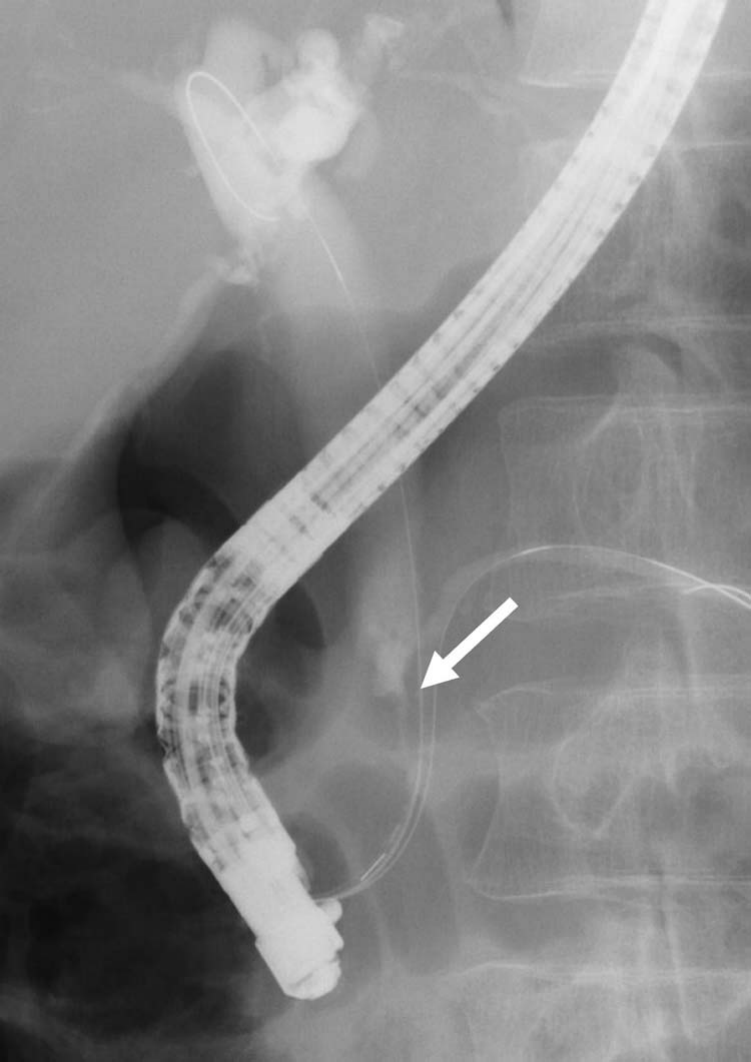
ERCP revealed irregular stricture of the distal CBD (arrow); brush cytology was performed at the same time.

Contrast-enhanced computed tomography (CT) revealed wall thickening and stricture of the distal common bile duct (CBD) (Fig. 1). Dilation of both the intrahepatic bile ducts and the common hepatic duct was also observed (Fig. 2). No lymph node or distant metastases were observed. Endoscopic retrograde cholangiopancreatography revealed irregular stenosis in the distal CBD (Fig. 3). Thereafter, biliary drainage and brush cytology were performed, and an adenocarcinoma was detected.

We performed subtotal stomach-preserving pancreaticoduodenectomy with lymph node dissection under a presumptive diagnosis of distal CBD cancer. The patient’s post-operative course was uneventful, and he was discharged on the 11th post-operative day.

**Figure 4:**
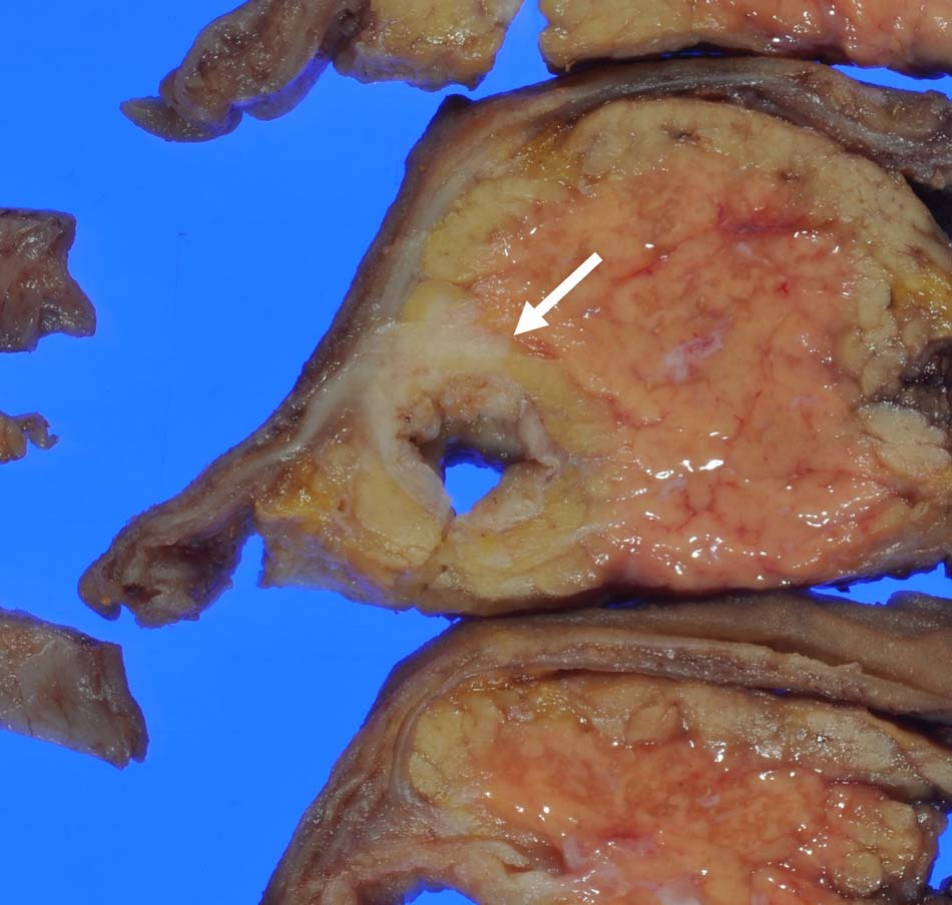
The surgical specimen showed a gray invasive nodular tumor measuring 19 × 18 × 15 mm at the distal CBD (arrow).

**Figure 5:**
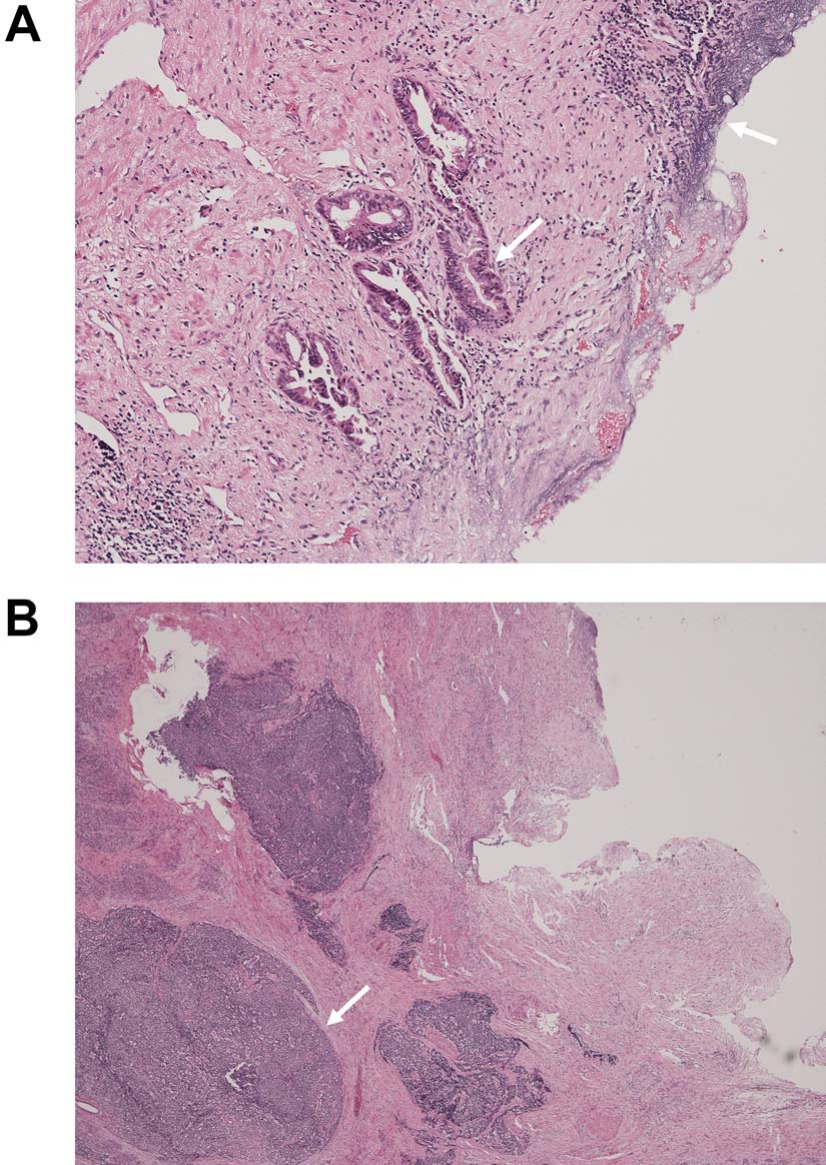
Pathological examination of the surgical specimen stained with hematoxylin and eosin; (**A**) well-differentiated adenocarcinoma at the CBD mucosa (arrow); (**B**) proliferation of small atypical cells forming follicular nests with invasion around the CBD wall (arrow).

**Figure 6:**
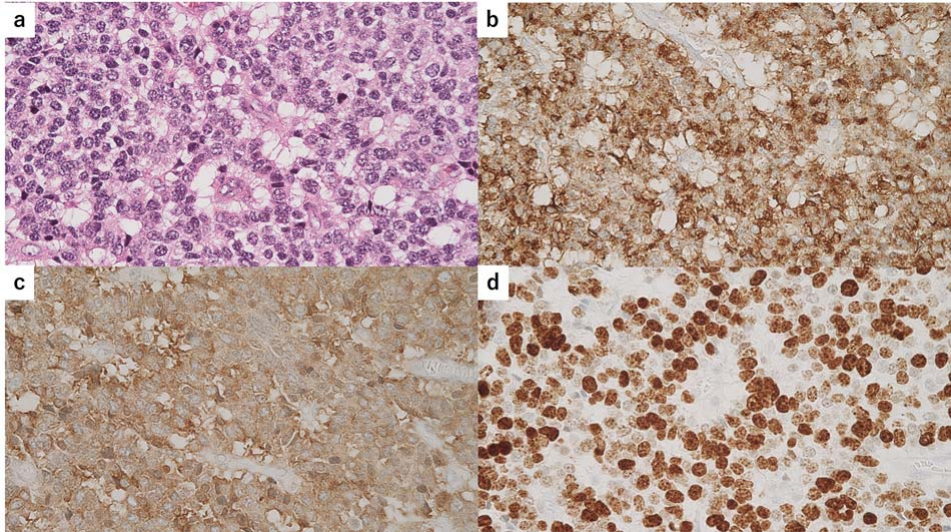
Immunohistochemical findings of the solid lesion of small atypical cells; (**a**) hematoxylin and eosin standing of the small atypical cells; (**b**) immunostaining for chromogranin A was positive; (**c**) immunostaining for synaptophysin was positive; (**d**) the Ki-67 index was ~80%.

Macroscopically, there was a gray invasive nodular tumor measuring 19 × 18 × 15 mm at the distal CBD (Fig. 4). Histopathological examination showed that the tumor comprised two components; well-differentiated adenocarcinoma of the CBD mucosa (20%) and proliferation of small atypical cells forming follicular nests with invasion around the CBD wall (80%) (Fig. 5a and b). The invasion extended to the pancreas and duodenal muscular layer. Immunohistochemical examination revealed that the solid proliferative lesions of small atypical cells were positive for chromogranin A and synaptophysin (Fig. 6a–c). The Ki-67 index was ~80% (Fig. 6d). No lymph node metastasis was detected. The patient was finally diagnosed with small-cell NEC (pT2N0M0, pStage I B) of the EHBD. R0 resection was achieved pathologically and there was no recurrence for 4 months after surgery.

## DISCUSSION

Neuroendocrine neoplasms (NENs) are tumors derived from neuroendocrine cells and are mostly detected in the digestive systems [2]. Conversely, NENs of EHBD are extremely rare and account for only 0.2–2.0% of primary sites [3]. According to the World Health Organization classification 2019, NENs of the digestive system are classified into neuroendocrine tumors and NECs by tumor differentiation, mitotic index and Ki-67 index based on pathological findings. Furthermore, NECs are classified as large- and small-cell NECs based on cell size.

NECs of EHBD account for only 0.19% of EHBD malignancies, and only a few cases have been reported [4]. Kamiya *et al*. reported 25 resected cases of EHBD NEC [5]. Similar to our case, most of these patients presented with obstructive jaundice as the primary symptom and were preoperatively diagnosed with EHBD cancer. Differentiation between EHBD cancer and EHBD NECs is very difficult because of the similarity in imaging findings. In addition, NECs of EHBD often contain adenocarcinoma components in the superficial layer of the bile duct mucosa, whereas the NEC component is found below the submucosal or deeper layer; therefore, the result of cytology or biopsy of the bile duct may mislead us to prediagnose cholangiocarcinoma [6]. Taken together, the preoperative diagnosis of EHBD is very difficult; thus, surgery is needed for a definitive diagnosis.

The prognosis of EHBD NEC is reportedly very poor. Over half of patients with gastroenteropancreatic NECs have distant metastasis when diagnosed initially, and EHBD NECs are relatively aggressive [7]. Even if the lesion is localized and radical surgery is performed, rapid recurrence is often observed in patients with EHBD NECs.

Therefore, appropriate strategies are required to reduce recurrence after curative surgery. The effectiveness of adjuvant chemotherapy has not been determined, but neoadjuvant chemotherapy has been reported to have some advantages over adjuvant chemotherapy [8, 9]. Many patients with NEC may have occult metastases at the time of surgery, which result in early recurrence and progression. Neoadjuvant chemotherapy can control occult metastases and prevent early recurrence after surgery [10]. Therefore, the accumulation of more patients who undergo surgery and studies on adjuvant or neoadjuvant chemotherapy are required. Chemotherapy is commonly considered for patients with distant metastasis at initial diagnosis or recurrence after surgery. Chemotherapeutic regimen usually involves cisplatin and etoposide. However, the response rates in patients with EHBD NECs are much lower than those in patients with pulmonary NECs [11]. Radiation therapy is also considered for case of localized recurrence or metastasis [12]. To improve the prognosis of EHBD NECs, surgery, chemotherapy and radiation therapy should be considered.

In conclusion, we encountered a rare resected case of small-cell NEC of the EHBD. A treatment strategy has not been established because of its rarity and difficulty in preoperative diagnosis. EHBD NECs have a very poor prognosis even if R0 resection is performed. Therefore, combined modality therapy, including surgery, chemotherapy and radiation therapy, should be considered. Further evaluation of the multimodal treatments for EHBD NECs is warranted.
